# Response of Mice and Ferrets to a Monovalent Influenza A (H7N9) Split Vaccine

**DOI:** 10.1371/journal.pone.0099322

**Published:** 2014-06-17

**Authors:** Yueqiang Duan, Hongjing Gu, Rui Chen, Zhongpeng Zhao, Liangyan Zhang, Li Xing, Chengcai Lai, Peirui Zhang, Zhiwei Li, Keming Zhang, Zhouhai Wang, Shaogeng Zhang, Xiliang Wang, Penghui Yang

**Affiliations:** 1 Beijing Institute of Microbiology and Epidemiology, State Key Laboratory of Pathogen and Biosecurity, Beijing, China; 2 Department of Pathogenic Biology and Medical Immunology, School of Basic Medical Sciences, Ningxia Medical University, Yinchuan, China; 3 302 Military Hospital, Beijing, China; Washington University School of Medicine, United States of America

## Abstract

In early spring 2013, the emergence of the influenza A (H7N9) virus in humans in Eastern China raised concerns of a new influenza pandemic. Development of a safe and effective H7N9 influenza vaccine is urgently needed. To this end, we first synthesized the hemagglutinin (HA) and neuraminidase (NA) genes of the influenza A (H7N9) virus A/AnHui/1/2013. Using reverse genetics, we rescued a reassortant virus (H7N9/PR8) that contained the HA and NA genes from wild-type H7N9 and six genes encoding internal proteins from the A/Puerto Rico/8/34 (PR8) virus. Next, the pathogenicity of the reassortant virus was evaluated both *in vivo* and *in vitro*. We found that the virus was non-pathogenic in mice and was stable after serial passaging in eggs. Furthermore, we found that a monovalent influenza A (H7N9) split vaccine prepared from the virus was immunogenic in mice and ferrets. When given intramuscularly, the vaccine (two doses of at least 15-µg) completely protected mice from normally lethal wild-type H7N9 virus challenge. In summary, our H7N9 vaccine, developed over a short time, is a potential candidate for further clinical evaluation and human use.

## Introduction

On 31 March 2013, the Chinese National Health and Family Planning Commission announced the occurrence of three cases of human infection with influenza A (H7N9) [1], [2]. As of 26 May 2013, a total of 135 human cases of influenza A (H7N9) infection had been reported, including 44 deaths. Until recently, infected patients continued to be diagnosed in Eastern China. To date, there is no indication that the virus can be transmitted between people, but the routes and mechanisms of transmission are being actively investigated in many laboratories worldwide.

Infection caused by influenza H7N9 virus is a zoonosis, and the animal reservoir of the virus is the global aquatic bird population [3], [4]. The epidemiological link between the presence of the H7N9 virus in poultry and the appearance thereof in humans in 2013 implied that influenza H7N9 viruses might be directly transmitted from birds to humans. Although control of the H7N9 outbreak was achieved by closing the live-bird markets of China, this episode has shown that our capability to deal with emerging influenza threats is inadequate. Therefore, in addition to active management measures, the development of an H7N9 vaccine is urgently needed to protect humans from the threat of an H7N9 influenza epidemic or pandemic [5].

Currently, several strategies are being used to develop vaccines that protect humans against influenza virus infection. Inactivated, subunit, and live attenuated vaccines are under development. As the influenza genome is segmented, laboratories in the WHO influenza network have generated a reassortant virus used for annual influenza vaccinations. This virus contains surface protein-encoding genes from the currently circulating influenza virus, but the remaining six genes, encoding internal proteins, are derived from PR8, a laboratory-adapted H1N1 strain. Thus, the reassortant virus has the antigenic characteristics of the circulating strain and the high-yield properties of PR8 [6], [7]. On 26 September 2013, the WHO recommended the use of and summarized the developmental status and availability of avian influenza A(H7N9) candidate vaccine viruses (http://www.who.int/influenza/vaccines/virus/candidates_reagents/a_h7n9/en/), but the experimental data on H7N9 influenza vaccines were incomplete.

In the present paper, we report on the creation of an H7N9 influenza vaccine candidate virus using reverse genetics. All experiments employed Vero cells approved by the WHO for the manufacture of human vaccines. Using an eight-plasmid system, we (rapidly) rescued a reassortant H7N9 influenza vaccine virus from such cells, and assessed the pathogenicity thereof both in vivo and in vitro. Next, the immunogenicity and protective efficacy of the candidate vaccine were evaluated in animal models. Our work shows that reverse genetics can be used to prepare for influenza pandemics that may threaten China.

## Materials and Methods

### Ethics Statement

All experiments using infectious influenza viruses, including animal studies with live viruses, were conducted under biosafety level 3 containment. Mice were housed at an appropriate temperature under a standard light/dark cycle. All animal experiments were conducted in accordance with the Guidelines for Animal Experiments of the Beijing Institute of Microbiology and Epidemiology and were approved by the Institutional Animal Care and Use Committee of that institute (approval no.: SYXK 2007-005). All infections and sample collections were performed under sodium pentobarbital anesthesia (60–80 mg/kg) and all efforts were made to minimize animal suffering.

### Viruses and Cells

The human influenza A H7N9 viruses (A/AnHui/1/2013), isolated in China in 2013 [8]–[10], and wild-type (wt) influenza PR8 (H1N1), were grown in the allantoic cavities of 10 day-old embryonated eggs, or MDCK cells. WHO-approved Vero cells (WHO-Vero; X38, p134) were sourced from the American Type Culture Collection (ATCC). Cells at passage 145 were used to generate the vaccine candidate. Plasmids containing the relevant PR8 genes have been described previously [11].

### Cloning of the Hemagglutinin and Neuraminidase Genes of the A/Anhui/1/2013 Virus

First, the hemagglutinin (HA) and neuraminidase (NA) genes of A/Anhui/1/2013 were synthesized by Sangon Biotech (Shanghai) Co. Ltd. The hemagglutinin gene was amplified by PCR using the primer pair (1): Bm-HA-1 (5′-TATTCGTCTCAGGGAGCAAAAGCAGGGG-3′) and Bm-HA-2 (5′ -ATATCGTCTCGTATTAGTAGAAACAAGGGTGTTTT-3′). The neuraminidase gene was amplified using the primer pair Ba-NA-1 (5′-TATTGGTCTCAGGGAGCAAAAGCAGGAGT-3′) and Ba-NA-2 (5′-ATATGGTCTCGTATTAGTAGAAACAAGGAGTTTTTT-3′). The PCR products were purified and cloned into the vector pHW2000, as described previously [11].

### Electroporation of Vero Cells

To obtain the reassortant H7N9/PR8 virus, six plasmid DNAs encoding internal proteins of PR8 were combined with the two plasmids encoding the surface antigens HA and NA of A/AnHui/1/2013. One day prior to electroporation, Vero cells at 90–100% confluence were split and seeded at a density of 9×10^6^ cells in MEM with 10% (v/v) FBS. The following day, the cells were trypsinized and resuspended in 50 ml of phosphate-buffered saline in a T25 flask. The cells were next pelleted and resuspended in 0.5 ml of OptiMEM 1 (Hyclone). A total of 5×10^6^ cells was added to a 0.4-cm (internal dimension) electroporation cuvette in a final volume of 400 µl of OptiMEM 1. Twenty micrograms of DNA, containing an equimolar mixture of the eight plasmids in a volume of no more than 25 µl, were next added to the cells in the cuvette. The DNA and cells were mixed gently by tapping and electroporated at 300 V and 950 µF in a Bio-Rad Gene Pulser II. The time constant was in the range 28–35 sec. The contents of the cuvette were again gently mixed by tapping and, 2–3 min after electroporation, 0.7 ml of MEM with 10% (v/v) FBS was added. The cells were again gently mixed by serial pipetting and divided between two wells of a six-well dish containing 2-ml MEM with 10% (v/v) FBS per well. Culture at 33°C followed. The medium was removed on each following day and stored. At 72 h post-electroporation, the final culture supernatants were harvested. Recovered virus was amplified in embryonated chicken eggs and stored at –80°C. Clarified supernatant (0.2-ml amounts) was injected into the allantoic cavities of 10 day-old embryonated chicken eggs. After 48 h of incubation at 35°C, allantoic fluids were harvested and tested for hemagglutination activity. The desired reassortant H7N9/PR8 virus was identified in allantoic fluid from the second passage in eggs.

### Pathogenicity Studies in Chickens

Eight 4-week-old White Rock chickens received intravenous injections (i.v) of 0.2 ml of 1∶10 dilution of stock virus. Oropharyngeal and cloacal swabs were collected at 3 days post-infection, and we did assays for the presence of virus by injection of 0.1 ml into all of four 10-day-old embryonated chicken eggs. Haemagglutination activity in the allantoic fluid of these eggs was evaluated after incubation at 35°C for 48 hours. On day 14 p.i., all surviving chickens were bled and sera were tested for evidence of sero-conversion by agar gel precipitin (AGP) and HAI assay.

### Pathogenicity in Mice

The MLD_50_ values of the H7N9/PR8 and PR8 transfectant viruses were determined by intranasal inoculation of groups of wild-type 6-week-old female BALB/c mice (Laboratory Animal Center, AMMS). Serial 10-fold dilutions of the viruses were administered to mice anesthetized with pentobarbital sodium. The mice were monitored daily for signs of disease to 14 days post infection. To measure viral replication in various tissues, groups of mice were intranasally inoculated with 10^6^ EID_50_ of the reassortant H7N9/PR8 vaccine, or wt virus, in volumes of 20 µl, respectively. On day 3 post-infection, animals were sacrificed and various tissues harvested and homogenized in MEM medium to 10% (w/v). Virus in these homogenates was titrated in Madin-Darby canine kidney (MDCK) cells, as described elsewhere.

### Preparation of an H7N9 Vaccine

The monovalent influenza A (H7N9) split virus vaccine was developed by the HengYe Biological Company, and the seed virus was prepared from the reassortant vaccine H7N9/PR8 virus. The vaccine was prepared in 10-day-old embryonated chicken eggs using techniques identical to those employed in current production of the trivalent inactivated vaccine against seasonal influenza. In brief, virus was harvested from egg cultures and inactivated with formaldehyde. The virus was concentrated, purified, and further sterilized by chromatography (on a Sepharose 4FF column) in a buffer containing Triton X-100. The monovalent influenza A (H7N9) split vaccine was produced on a pilot scale using Good Manufacturing Practices. The vaccine contained all proteins of the viral particle, and the major component was HA (about 30% of total protein). The four experimental vaccines produced were split virus products containing 7.5-, 15-, 30-, and 45-µg HA, and were prepared using WHO-approved protocols [12]. The vaccines were stored at 4°C prior to use.

### Immunogenicity and Efficacy of the H7N9 Split Vaccines

Groups of six-week-old BALB/c mice (20 per group) and ten-week-old ferrets (Angora LTD, Jiangsu, China) (8 per group) were immunized intramuscularly with two doses (20 or 200 µl; mice and ferrets, respectively) of vaccine containing 7.5, 15, 30, or 45 µg of HA, at 14-d intervals, and sera were collected 2 weeks after priming and 2, 4, and 8 weeks after boosting. The serum titers of hemagglutination-inhibition (HI) antibodies were measured using standard methods; 4 HA units of the two viruses were mixed, in the wells of 96-well plates, with 0.5% (v/v) turkey erythrocytes. Plates used to detect H7N9 influenza-specific IgG, IgG1, and IgG2a in sera were coated using a solution of 5 µg/ml inactivated influenza virions. Samples were serially diluted in fivefold increase in PBS containing 1% (w/v) bovine serum albumin (Serva) and added to coated plates. Bound antibodies were detected using 1∶10,000 dilutions of goat anti-mouse IgG, IgG1, and IgG2a (Sigma), conjugated to horseradish peroxidase (HRP). Plates were stained with the TMB substrate (Sigma) and OD values read at 450 nm. Each sample was assayed in duplicate.

Two weeks after the second dose of the H7N9 split vaccine, 15 mice in each group were challenged intranasally with (wt) AnHui H7N9 virus (5.0×10^3^ TCID_50_ pfu; equivalent to 4.0×LD_50_). Prior to virus instillation, mice were anesthetized with pentobarbital sodium. After challenge, all mice were observed for signs of illness and weighed daily over the next 14 days. Mice identified as moribund or who lost >25% of body weight were considered to have attained an experimental endpoint and were humanely euthanized. On day 3 post-challenge, mice (five per group) were euthanized and lung tissue collected for virus titration (to evaluate protection from infection). Mice were humanely euthanized via cervical dislocation and carefully observed and palpated to verify the absence of respiration and heartbeat.

### Statistical Analysis

All data were analyzed using the GraphPad Prism 5.0 software. Antibody and virus titers were compared using the two-tailed *t*-test. Survival rates were evaluated by Kaplan-Meier analysis.

## Results

### Generation of the H7N9/PR8 Transfectant Virus

The first step in production of an H7N9 influenza vaccine was rescue of the candidate vaccine virus. The HA and NA genes of A/AnHui/1/2013 were artificially synthesized and individually cloned into the vector pHW2000. The two resulting plasmids, and six plasmids encoding the remaining proteins of PR8, were transfected into Vero cells to rescue the vaccine virus, termed H7N9/PR8. Approximately 60 h after transfection, a cytopathic effect was evident when Vero monolayers were examined. The rescued virus grew to high titers on subsequent culture in eggs (hemagglutination titers of 1∶512–1∶1,024). After two passages, the rescued virus was fully sequenced and was identical as expected.

### Properties of the Reassortant H7N9/PR8 Virus

**Figure 1:**
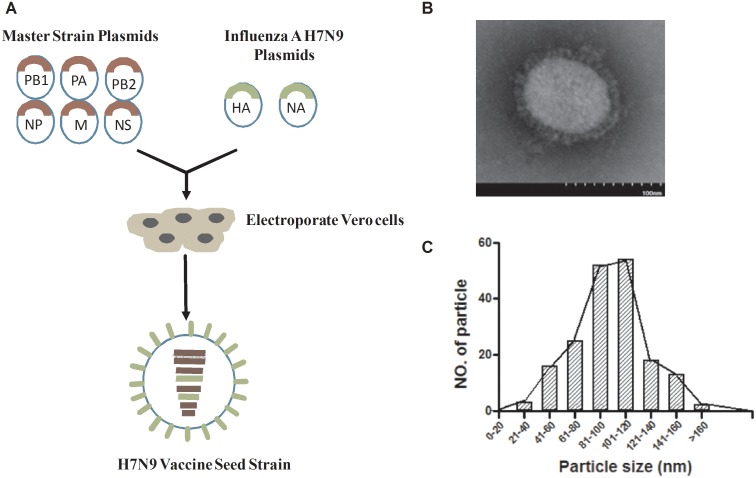
Production of the H7N9/PR8 influenza vaccine candidate using reverse genetics, and the size distribution and shapes of the virus particles. (A) Preparation of the H7N9 vaccine seed strain using eight-plasmid reverse genetics. (B) Electron micrograph of recombinant H7N9/PR8 virus particles. (C) Most (73%) of 200 H7N9/PR8 virus particles were 80–120 nm in diameter.

As shown in Figure 1A, we used reverse genetics to generate a 6∶2 reassortant virus, H7N9/PR8, containing the HA and NA genes from the wt H7N9 AnHui virus and six genes encoding internal proteins of the PR8 virus (we used plasmids pHW191 to pHW198 to this end). The reassortant H7N9/PR8 virus grew to an HA titer of 1∶512-1,024 and an infectivity of 8.5 log_10_EID_50_/ml in eggs. The H7N9/PR8 and PR8 transfectant viruses could form plaques on MDCK cells in the presence of 1 µg/ml TPCK trypsin (Sigma), whereas the wt H7N9 virus formed plaques with similar efficiency in either the presence or absence of TPCK-trypsin (data not shown). Recombinant H7N9/PR8 viral particles were examined by electron microscopy, which revealed spherical structures approximately 80–120 nm in diameter, with the characteristic lipid membrane bilayer on the outer surface (Figs. 1B and C). The H7N9/PR8 virus is therefore a typical influenza virus in terms of both morphology and size, suggesting that no major structural change was created during construction. We also found that the H7N9/PR8 virus was stable on continued serial passage in embryonated eggs and no change in the nucleotide sequences encoding HA, NA, and NP was evident after 12 passages. As seen in Table 1, intravenous administration of a 1/10 dilution of H7N9/PR8 did not result in any signs of infection in chickens, and we were unable to detect any virus in swabs of oropharyngeal or cloacae from inoculated animals. In addition, no apparent change in virus titer developed upon repeat passage (data not shown).

**Table 1:** Pathotyping and replication of intravenously administrated H7N9/PR8 transfectant virus in chickens^a^.

Virus	Mortality(death/total)	Virus isolation from swabs^b^				Seroconversion^c^(seroconverted/total)
		Oropharyngeal		Cloacal		
		Shedding/total	Titers	Shedding/total	Titers	
PR8	0/8	1/8	1.2±0.08	5/8	3.2±0.02	7/8
H7N9/PR8	0/8	0/8	≤1.0	0/8	≤1.0	3/8

a Groups of chicken were infected i.v. with 0.2 ml of 1∶10 dilution of stock virus.

b Oropharyngeal and Cloacal swabs were collected for virus titration on day 3 after inoculation. A mean value of 1.0 was considered for calculation if virus was not isolated from a swab.

c Chickens were bled at 2 weeks after inoculation. Sero-conversion was confirmed and analyzed.

### Pathogenicity and Replication in Mice

To explore the pathogenicity of the viral vaccine, we compared the properties of the H7N9/PR8 vaccine and (wt) AnHui virus in mice. The influenza PR8 virus is a highly passaged laboratory strain that is known to be lethal for mice. As shown in Table 2, wt AnHui influenza H7N9 virus replicated in the lungs of mice. The PR8 transfectant virus and the wt H7N9 virus were lethal for mice (MLD_50_ values of 3.2 Log_10_ EID_50_ and 3.8 log_10_ EID_50_, respectively) but the H7N9/PR8 transfectant virus was not lethal (MLD_50_>6.0 log_10_ EID_50_). The H7N9/PR8 virus replicated to lower titers than did the wt H7N9 or PR8 transfectant virus in lung tissues of mice. Virus replication was undetectable in the brains, spleens, liver and kidneys of mice when administrated with H7N9/PR8, *wt* H7N9 Anhui01 virus or PR8-transfectant viruses. These data indicated that the reassortant H7N9/PR8 vaccine virus was attenuated for mice.

**Table 2:** Pathogenicity and replication of a reassortant H7N9/PR8 virus in BALB/c mice^a^.

Virus	Mean virus titer (log_10_TCID_50_) on day 3 post-inoculation				
	Lung	Brain	Spleen	Liver	Kidney
PR8	5.7±0.12	≤1.0	≤1.0	≤1.0	≤1.0^c^
AnHui 01	5.4±0.11	≤1.0	≤1.0	≤1.0	≤1.0
H7N9/PR8	4.6±0.10^b^	≤1.0	≤1.0	≤1.0	≤1.0

a BALB/c mice were inoculated intranasally with 10^6^ EID_50_ of the indicated viruses and euthanized 3 days later. Virus titers in lung, brain, spleen, and liver tissue were determined by titration in MDCK cells. Geometric mean titers ± SDs are shown.

b p<0.01 compared with the tiers in the corresponding organs of the AnHui 01 or PR8-inoculated mice.

c The lower limit of detection was 10 TCID_50_/g of tissue.

### Immunogenicity in Mice and Ferrets

**Figure 2:**
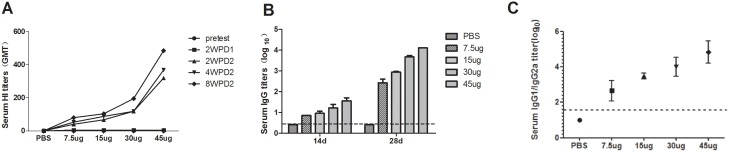
Antibody responses to the H7N9/PR8 split vaccine in mice. Groups of 20 BALB/c mice were immunized intramuscularly at weeks 0 and 2 with 7.5, 15, 30, or 45 µg (HA levels) of the H7N9/PR8 split vaccine. (A) HI antibody responses to the (wt) AnHui virus after vaccination as described above. Serum samples were collected on day 0; 2 weeks after priming; and 2, 4, and 8 weeks after boosting. (B) Serum IgG titers against the (wt) AnHui virus measured 2 weeks after both the first and second doses of vaccine. (C) The ratios of serum IgG1 to IgG2a titers against the (wt) AnHui virus, calculated 2 weeks after the second dose of vaccine. The data are presented as means ± SDs of the data from three experiments, each performed in duplicate. HI, hemagglutination inhibition; PBS, phosphate-buffered saline. 2WPD1: 2 weeks post-vaccination with dose 1. 2WPD2: 2 weeks post-vaccination with dose 2; 4WPD2: 4 weeks post-vaccination with dose 2; 8WPD2: 8 weeks post-vaccination with dose 2.

The immunogenicity of the reassortant H7N9/PR8 virus was assessed in mice 2 weeks after the first immunization with the H7N9/PR8 split vaccine. Five groups of BALB/c mice were intramuscularly immunized at weeks 0 and 2, with vaccine containing 7.5, 15, 30, or 45 µg of the HA of the H7N9/PR8 split vaccine. By 14 days after the second vaccination, the titers of HI antibodies had increased sharply in each group. The HI titers against Anhui virus increased in a dose-dependent manner on 2, 4, and 8 weeks after administration of the second of two doses of vaccine (Fig. 2A). Similarly, the total IgG titers of mice immunized with the H7N9/PR8 split vaccine were significantly higher after boosting compared to after priming, and the titers were vaccine dose-dependent (Fig. 2B). As shown in Figure 2C, the IgG1 to IgG2a ratios of each vaccinated group were significantly higher than that of the PBS group after boosting with the H7N9 influenza virus. These results showed that H7N9/PR8 was highly immunogenic and preferentially induced a T_H_2-type cellular immune response in BALB/c mice.

**Figure 3:**
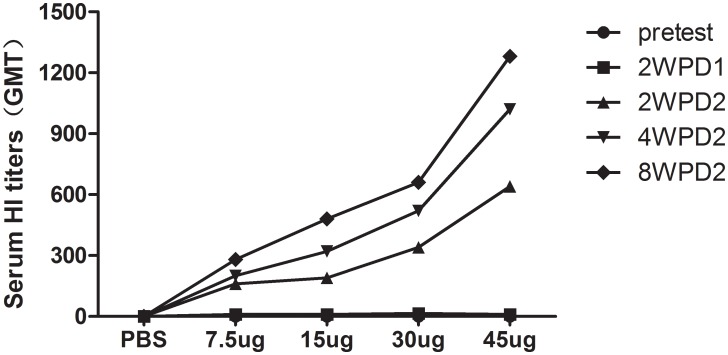
Antibody responses of ferrets immunized with H7N9/PR8 virus. HI antibody responses to the (wt) AnHui virus after intramuscular vaccination with 7.5, 15, 30, or 45 µg (HA levels) of H7N9/PR8 split vaccine, in 200-µl volumes. Serum samples were collected on day 0; 2 weeks after priming; and 2, 4, and 8 weeks after boosting. 2WPD1: 2 weeks post-vaccination dose 1. 2WPD2: 2 weeks post-vaccination dose 2; 4WPD2: 4 weeks post-vaccination dose 2; 8WPD2: 8 weeks post-vaccination dose 2. The lower limit of detection is indicated by the dashed horizontal line.

As was also observed in mice, HI antibody responses were evident in all ferrets at 2, 4, and 8 weeks after two vaccinations. As was seen in Table 3, two doses of the H7N9/PR8 split vaccine induced GMT titers of 160, 190, 340 and 640, respectively, against homologous AnHui virus in the 7.5-, 15-, 30-, and 45-µg HA groups by 2 weeks after the second immunization. Furthermore, at 8 weeks after boosting, the GMT titers against AnHui virus were 280, 480, 660 and 1280, respectively, in the four groups (Fig. 3). Meanwhile, no sign of clinical disease or weight loss was observed in vaccinated ferrets to 14 days after intramuscular vaccination. In addition, this vaccination was well tolerated and not overt clinical symptoms were identified. These results showed that two intramuscular vaccinations with the split H7N9 vaccine prepared from the H7N9/PR8 virus were immunogenic and caused no apparent adverse events in any experimental group of either mice or ferrets. Ferrets developed a more pronounced antibody response than did mice.

**Table 3:** Immunogenicity of a H7N9 split vaccine prepared from the H7N9/PR8 transfectant virus.

Immunogen^a^	HAI antibody titer^b^ (GMT)			
	Mice		Ferrets	
H7N9/PR8 spilt vaccine	7.5 µg	80	160	
	15 µg	104	190	
	30 µg	195	340	
	45 µg	320	640	
PBS	–	<10	<10	

a Groups of mice and ferrets received two doses of 7.5, 15, 30 or 45 µg monovalent influenza A (H7N9) split vaccine or PBS i.m in 14 days intervals.

b Serum samples were collected and assayed at 2 weeks after boost.

### Protection of Mice from Challenge with Influenza A H7N9 Viruses

**Figure 4:**
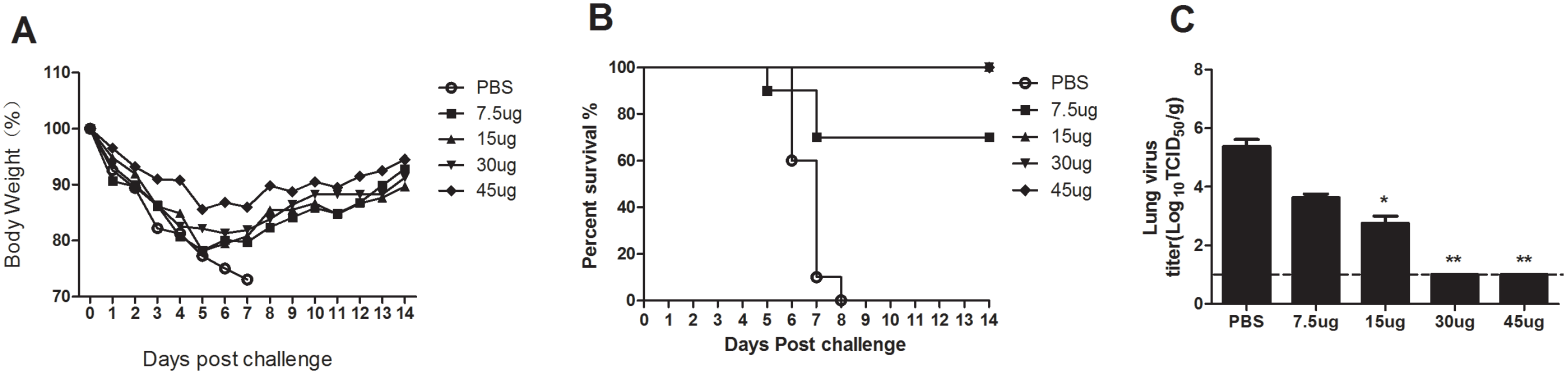
Efficacy of the H7N9/PR8 split vaccine in mice. Mice that had been intramuscularly immunized twice with phosphate-buffered saline (control); or 7.5, 15, 30, or 45 µg (HA levels) of influenza A H7N9 split vaccine, were challenged with a (normally) lethal dose of (wt) A/AnHui/1/2013 (H7N9) virus 2 weeks after the boost. At day 3 after challenge, mice were euthanized and lung tissues collected. (A) Lung virus titers were determined by calculation of TCID_50_ values in MDCK cells. Weight changes in (B) and survival rates of (C) mice immunized with 7.5, 15, 30, or 45 µg (HA levels) of the influenza A H7N9 split vaccine, and subsequently challenged with a (normally) lethal dose of (wt) A/AnHui/1/2013 (H7N9) virus. *p<0.01 and **p<0.001 compared to PBS-immunized mice. The lower limit of detection is indicated by the dashed horizontal line.

To further evaluate the protective efficacy of the H7N9/PR8 split vaccine, immunized mice were challenged intranasally with live AnHui virus. By 2 weeks after the second immunization with various doses of the H7N9/PR8 split vaccine, the titers of HI antibodies against the homologous AnHui virus had increased significantly over pretest values. Following immunization with two doses of the H7N9/PR8 split vaccine, groups of 15 mice were i.n. challenged with 5.0×10^3^ TCID_50_ (equivalent to 4.0×LD_50_) of live AnHui H7N9 virus. Mice receiving 15, 30, and 45 µg of vaccine HA were highly resistant to challenge with live AnHui virus; all mice survived for more than 14 days after (normally) lethal challenge and body weight recovered quickly (by 7 days post-challenge). In contrast, the survival rate of the 7.5-µg HA vaccinated group was only 70% (Figs. 4A and B), and mock-immunized mice died between days 6 and 9 post-challenge.

The efficacy of the H7N9/PR8 split vaccine in preventing viral replication was evaluated on day 3 post-challenge. Mice (five per group) were euthanized and tissues were collected for virus titration; the remaining mice of each group were observed for a further 2 weeks. As shown in Figure 4C, the H7N9/PR8 split vaccine afforded partial protection, when given at 7.5 and 15 µg (the HA levels), against pulmonary replication of the (wt) AnHui virus to day 3 post-challenge. A significant decrease (p<0.001) in viral load was evident in mice immunized with the H7N9/PR8 split vaccine at 30 and 45 µg HA, compared with unvaccinated controls, consistent with enhanced viral clearance by the vaccinated animals. Thus, the monovalent H7N9/PR8 split vaccine protected against a lethal dose of wt A/AnHui/1/2013 and inhibited pulmonary virus replication.

## Discussion

The rapid development of potential candidate vaccines against the 2013 influenza A (H7N9) outbreak began when the potential for disaster was apparent. It remains possible that a pandemic may arise this winter and rapid production of an appropriate vaccine may be needed to protect against H7N9 outbreaks even in 2014 [13]. Due to the need to rapidly manufacture new vaccines in response to the influenza A (H7N9) virus, a unique set of regulatory requirements must be applied to the development, testing, and batch release of influenza H7N9 vaccines. Most influenza vaccines are the whole virus vaccines, split virus vaccines, and subunit vaccines. In split virus vaccines, the virus has been disrupted by a detergent. While in subunit vaccines, HA and NA have been further purified by removal of other internal viral components which is related to immunogenicity and protective efficacy of influenza vaccine [14], [15]. Reverse genetics, used to generate influenza virus from cells co-transfected with plasmids carrying individual influenza virus genes, has been used in vaccine development since 1996 [6], [16]–[18]. As reported, reverse genetics to generate reassortant viruses containing HA and NA of pdmH1N1 while the remaining six internal genes are derived from the A/PR/8/34 (H1N1) might be hampered by some, yet to be defined, inherent factors of the virus [19]. So far, a number of reverse genetics-derived 6∶2 reassortant viruses have been reported. Most, if not all, contain specific changes on HA compared with their parental strains in order to grow efficiently in eggs [20]. Herein, the influenza A H7N9 virus A/AnHui/1/2013 was isolated from a confirmed H7N9 patient, by culture in MDCK cells, on March 31, 2013 [1], [2], [8], [21]. Herein, using reverse genetics, we produced an H7N9 candidate vaccine that contains the HA and NA genes of the AnHui (wt) virus and six genes encoding internal proteins of the PR8 virus. A PR8-based vaccine can be parenterally administered in an inactive form, and vaccine manufacturers have more experience with PR8-based reassortant vaccines than with other vaccines.

In this paper, we report our successful efforts to develop an H7N9 vaccine that resembles the currently licensed seasonal influenza A vaccines. The H7N9/PR8 virus has many characteristics that are desirable in an H7N9 vaccine candidate. The absence of pathogenicity and infectivity for chickens should allow manufacture of an egg-based vaccine. Herein, we investigated the properties of the H7N9/PR8 candidate vaccine in chickens, mice and ferrets. Our candidate vaccine virus grows efficiently in eggs (to 10^8.5^ EID_50_/ml). The vaccine is not pathogenic for chickens, mice, or ferrets, and failed to replicate in the brain, spleen, liver, or kidney of infected animals. The level of replication of the reassortant H7N9/PR8 virus in the lungs of mice was lower than those of the PR8 or (wt) Anhui virus. A monovalent inactivated split vaccine prepared from the H7N9/PR8 virus was immunogenic and protected mice from subsequent (normally) lethal live AnHui virus challenge. Taken together, our findings demonstrate that reverse genetics is compatible with putting H7N9 HA and NA genes on a PR8 background for vaccine production and warrant human testing of the H7N9/PR8 virus as a potential candidate vaccine in a clinical context. Of course, additional research into the immunogenicity and protective efficacy of the H7N9/PR8 virus is required in ferrets, monkeys, and humans.

The influenza H7N9 virus-like particle (VLP) vaccine, consisting of full-length, unmodified hemagglutinin (HA) and neuraminidase (NA) from A/AnHui/1/2013, and the matrix 1 (M1) protein from A/Indonesia/05/2005 (H5N1), was immunogenic and protected mice from lethal challenge with wt virus [22], [23]. Others have shown that an H3 stalk-based chimeric hemagglutinin, designed to serve as a universal influenza virus vaccine, protected mice against H7N9 challenge [24]. In line with the report of De Groot (5), two doses of inactivated H7N9 vaccine (rather than one dose) were required, because H7N9 was exhibited poor immunogenicity (as predicted). We found that two or more 15 µg doses (in HA terms) of the H7N9/PR8 split vaccine was more effective than one dose to completely protect against a lethal challenge with the wt Anhui virus, as measured by less body weight loss and an increase in survival rate. As shown in Figure 3, measurement of viral titers in lung tissues showed that the vaccine could protect mice from pulmonary virus replication. Overall, our data suggest that two 15-µg (HA) doses of the H7N9/PR8 split vaccine may be effective in humans.

Influenza infection in ferrets has several features resembling human infection in terms of clinical signs and immunity, rendering the ferret an appropriate model in which to assess the efficacy of influenza vaccines [25], [26]. In the present paper, the reassortant influenza A H7N9/PR8 virus was not lethal in mice or ferrets. As shown in Figure 3, we found that the signs of illness were not observed in ferrets after vaccination and ferrets mounted a more active antibody response than did mice. Also, the high HI titers attained in the sera of vaccinated ferrets were sustained for a long period, extending even to 8 weeks after the second immunization. Thus, it is crucial to assess the immune response to influenza A H7N9 vaccines in ferrets, when seeking to develop appropriate models for the preclinical evaluation of human vaccines. We did not address the efficacy or protective effect of the influenza A H7N9 split vaccine in ferrets in the present study, and additional work is required to determine the efficacy, immunogenicity, safety, toxicity, and other pharmacological parameters of the influenza A H7N9 vaccine in appropriate animals before such effects can be evaluated in primates and humans.

It is difficult to predict the influenza subtype that may trigger a pandemic. Thus, it is important to be able to prepare influenza virus vaccines in a short period of time and to evaluate safety and immunogenicity in clinical studies prior to development of a pandemic. Overall, our results indicate that we have the technical expertise and virus stockpiles enabling us to respond rapidly to outbreaks via production of a safe candidate influenza vaccine virus. Of course, much remains to be done before such viruses can be used to protect humans against pandemic influenza. We believe that our work on the research and development of the 2013 influenza A (H7N9) candidate vaccine virus shows that is both challenging and possible to respond to the threat of an influenza pandemic when major efforts are made by vaccine manufacturers and clinical trial organizations.
